# Validated LC-ESI-MS/MS method for the determination of ivosidenib in 10 μL mice plasma: application to a pharmacokinetic study

**DOI:** 10.5599/admet.648

**Published:** 2019-03-05

**Authors:** Sreekanth Dittakavi, Rakesh Kumar Jat, Sadanand Rangnathrao Mallurwar, Ravi Kumar Jairam, Ramesh Mullangi

**Affiliations:** 1 Drug Metabolism and Pharmacokinetics, Jubilant Biosys, Industrial Suburb, Yeshwanthpur, Bangalore-560 022, India; 2 Institute of Pharmacy, Shri Jagdish Prasad Jhabarmal Tibrewala University, Jhunjhunu-Churu Road, Chudela, Jhunjhunu-333001, Rajasthan

**Keywords:** Ivosidenib, LC-MS/MS, method validation, mice plasma, pharmacokinetics, bioavailability

## Abstract

A simple, selective and rapid LC-ESI-MS/MS method has been developed and validated for the quantification of ivosidenib in mice plasma using warfarin as an internal standard (I.S.) as per regulatory guideline. Sample preparation was accomplished through a simple protein precipitation process. Chromatography of ivosidenib and the I.S. was achieved on an Atlantis dC_18_ column using an isocratic mobile phase comprising 0.2 % formic acid in water and acetonitrile (25:75, v/v) delivered at a flow rate of 1.0 mL/min. LC-MS/MS was operated under the multiple reaction-monitoring mode (MRM) using the electrospray ionization technique in positive ion mode and the transitions of m/z 583.1→186.1 and m/z 309.2→251.3 were used to quantitate ivosidenib and the I.S, respectively. The total chromatographic run time was 2.0 min. Linearity was established in the concentration range of 1.10-3293 ng/mL (r^2^>0.99). The intra- and inter-day accuracy and precision for ivosidenib in mice plasma were in the range of 5.72-9.91 and 5.90-10.7 %, respectively. Ivosidenib was found to be stable on bench-top for 6 h, up to three freeze-thaw cycles, in in-injector for 24 h and for one month at -80 °C. The applicability of the validated method has been demonstrated in a mice pharmacokinetic study. Following intravenous (2 mg/kg) and oral (5 mg/kg) administration of ivosidenib to mice, concentrations were quantifiable up to 24 and 48 h, respectively. The bioavailability was 61 %.

## Introduction

**Figure 1.**
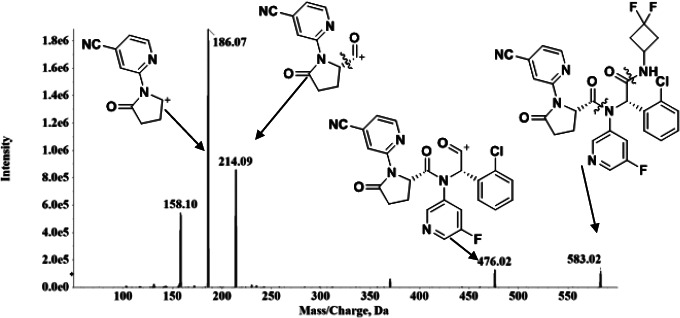
Fragmentation pattern of ivosidenib

Isocitrate dehydrogenase isoforms (IDH1 and IDH2/3 located in cytoplasm and mitochondria, respectively) play a key role in normal cell growth and regulation. All three isoforms are responsible for the production of nicotinamide adenine dinucleotide phosphate (NADPH) from NADP^+^ by catalyzing the oxidative decarboxylation of isocitrate to α-ketoglutarate (α-KG) [1]. Recent investigations discovered that IDH1/2 mutations in 20 % of acute myeloid leukemia (AML) population and rarely in breast, lung and prostate cancers [2]. Targeting mutant IDH1/2 proved to be an option for the treatment of AML as a targeted therapy alone or in combination with other antileukemic agents [3]. Ivosidenib (AG-120, Tibsovo®; Fig. 1), is a novel, first-in-class, oral small molecule, which selectively and reversibly inhibits mIDH1 (IC_50_: 12 nM) without any IDH2 inhibition (at micromolar concentration) along with good cellular potency. It showed tumor regression in mice xenograft model of IDH-mutated AML at 50 and 150 mg/kg doses [4]. Recently, FDA approved ivosidenib as an orphan drug for relapsed or refractory IDH1 mutant AML patients as detected by an FDA-approved test [5]. In humans as a monotherapy it is administered at a daily dose of 500 mg. Following oral administration *C*_max_ (maximum concentration in plasma) attained at ~3 h (*T*_max_). It has shown less than dose proportional increase in AUC (area under concentration time curve) and *C*_max_ from 200-1200 mg. Unchanged ivosidenib is majorly (92 %) seen in plasma. Ivosidenib is 92-96 % bound to plasma proteins. CYP3A4 mainly involved in its metabolism (N-dealkylation and hydrolysis). It is eliminated as parent up to 67 and 10 % in the feces and urine, respectively. The oral bioavailability was found to be 57 % [5].

LC-MS/MS is a powerful tool for rapid quantitation of drugs in various biological matrices with great accuracy and sensitivity with a shorter run time. Preclinical pharmacokinetics has a great influence on the development and investigation of potential preclinical candidates with better advice for the further drug design. Pharmacokinetic studies in preclinical species can both provide toxicological and clinical information and direct optimization of drug candidates; as a result, they play necessary parts in drug discovery and development. To date there is no LC-MS/MS method reported for quantification of ivosidenib in any biological matrix. In this paper, we are reporting the development and validation of a sensitive, selective and rapid LC-ESI-MS/MS method for quantitation of ivosidenib in mice plasma. The validated method was successfully used in a mice pharmacokinetic study following intravenous and oral administration of ivosidenib at 2.0 and 5.0 mg/kg dose, respectively.

## Materials and methods

### Materials

Ivosidenib (purity: ≥98 %) was purchased from Angene International Limited, England, UK. Warfarin (I.S; purity: 98.7 %) was purchased from Sigma-Aldrich, St. Louis, USA. LC-MS-grade acetonitrile and methanol were purchased from JT Baker, Avantor Performance Materials, PA, USA. Formic acid was purchased from Avra Synthesis Pvt. Ltd, Hyderabad, India. The control Balb/C mice K_2_.EDTA plasma sample was procured from Animal House, Jubilant Biosys.

### Instrumentation and chromatographic conditions

A Shimadzu HPLC Prominence (Shimadzu, Japan) coupled with Sciex 6500 triple quadrupole (Sciex, Redwood City, CA, USA) mass spectrometer was used for all analyses. The instrument was controlled using Analyst software (version 1.6.2). Ivosidenib and the I.S. were eluted using an isocratic mobile phase, which is a mixture of 0.2 % formic acid in water and acetonitrile (25:75, v/v) and chromatographed on an Atlantis dC_18_ column (50 0 4.6 mm, 3 μm) maintained at 40 ± 1 °C. The flow-rate was 1.0 mL/min. The mass spectrometer was operated in the multiple reaction mode (MRM) with positive electrospray ionization for the quantitation of ivosidenib and the I.S. Ionization was conducted by applying a voltage of 5500 V and source temperature was set at 500 °C. For analyte and the IS the optimized source parameters viz., curtain gas, GS1, GS2 and CAD were set at 55, 55, 65 and 10 psi. The compound parameters viz., declustering potential (DP), entrance potential (EP), collision energy (CE) and collision cell exit potential (CXP) 78, 10, 36, and 5 V for ivosidenib and 61, 10, 20, and 18 V for the IS. The mass transition *m/z* (precursor ion → product ion) 583.1→186.1 and 309.2→251.3 were monitored for ivosidenib and the I.S, respectively. Quadrupole Q1 and Q3 were set on unit resolution. The dwell time was 100 msec.

### Preparation of stock and standard solutions

Primary stock solutions of ivosidenib for preparation of calibration curve (CC) and quality control (QC) samples were prepared from separate weighing. The primary stock solution of ivosidenib (1144 μg/mL) and the I.S. (1000 μg/mL) were made in methanol:water (80:20, v/v) and DMSO, respectively. The primary stock solution of ivosidenib and the I.S. were stored at -20°C, which were found to be stable for 45 days. Ivosidenib primary stock solution was successively diluted in methanol:water (80:20, v/v) to prepare secondary stock and working stock solutions, which were used to prepare CCs and QCs. Working stock solutions were stored approximately at 4 °C for ten days. A working stock of the I.S. solution (100 ng/mL) was prepared in methanol. Samples for the determination of precision and accuracy were prepared by spiking control mice plasma in bulk with ivosidenib at appropriate concentrations: 1.10 ng/mL (lower limit of quantitation quality control, LLOQ QC), 3.29 ng/mL (low quality control, LQC), 1715 ng/mL (medium quality control, MQC) and 2607 ng/mL (high quality control, HQC) and were stored at -80 ± 10 °C until analysis.

### Sample preparation

To an aliquot of 10 μL plasma, 500 μL of methanol containing 100 ng/mL I.S. was added and vortex mixed for 2 min. Thereafter the contents were centrifuged for 5 min at 14,000 rpm in a refrigerated centrifuge (Eppendorf 5424R) maintained at 5 °C. Clear supernatant (200 μL) was transferred into vials and 2.0 μL was injected onto LC-MS/MS system for analysis.

### Validation procedures

The validation experiments were performed in accordance with the US Food and Drug Administration guideline [6].

The selectivity of the method was determined by the presence of interfering peaks from six individual drug-free mice plasma samples at the retention times of ivosidenib and the I.S. The auto-injector carryover was determined by injecting the highest calibration standard, followed by injection of blank plasma samples.

The LLOQ was determined as the concentration that has a precision of <20 % of the relative standard deviation and accuracy between 80-120 % of the theoretical value. The response of the blanks was then compared to that of the LLOQ.

Recovery was determined at LQC and HQC. Recovery for ivosidenib was calculated by comparing the mean peak response of pre-extraction spiked samples (spiked before extraction; n=6) to that of neat samples (n=6) at each QC level. Matrix effect was assessed by comparing the analyte mean peak areas at LQC and HQC concentration after extraction with the mean peak areas for post extracted blank plasma samples spiked with analyte at equivalent concentrations. Recovery and matrix effect for the I.S. was assessed at single concentration (100 ng/mL).

The precision and accuracy of the method were evaluated by measuring the four QC samples (LLOQ QC, LQC, MQC and HQC), which were prepared on each validation day (n=6 each). Inter-day precision was assessed on four separate days. Inter- and intra-day precisions were determined by calculating percent relative standard deviation (%RSD) that should be ≤15 % for all QCs except for LLOQ QC where it should be ≤20 %. The inter- and intra-day accuracy expressed as percent relative error (%RE) was calculated by comparing the measured concentration with the nominal value and deviation was limited within ±15 % except for LLOQ QC where it should be ≤20 %.

Freeze-thaw stability following three freeze-thaw cycles was evaluated (one day duration between each freeze-thaw cycle and ivosidenib spiked plasma samples were stored in -80 ± 10 °C between freeze/thaw cycles). The plasma samples were thawed at room temperature for 1 h and returned to the freezer. Bench-top stability was assessed by analyzing samples that had been kept at ambient temperature (25 ± 1 °C) for 6 h. Long-term stability was performed by analyzing samples that had been stored at -80 ± 10 °C for 30 days. The stability of ivosidenib and the I.S. in the injection solvent was determined periodically by injecting replicate preparations of processed plasma samples for up to 24 h (in the auto-sampler at 5 °C) after the initial injection. The peak-areas of the analyte and the I.S. obtained at initial cycle were used as the reference to determine the stability at subsequent points. These stability samples were processed and quantified against freshly spiked calibration curve standards along with freshly spiked QC samples. Samples were considered to be stable if assay values were within the acceptable limits of accuracy (±15 % RE) and precision (≤20 % RSD).

To evaluate the effect of dilution over the calibration range, the accuracy and precision of dilution control samples at 9881 ng/mL (n=6; 3 times of the ULOQ) were assessed by performing a 5- and 10-fold dilution.

### Pharmacokinetic study in mice

All the animal experiments were approved by Institutional Animal Ethical Committee (IAEC/JDC/2017/133). Male Balb/C mice (n=24) were procured from Vivo Biotech, Hyderabad, India. The animals were housed in Jubilant Biosys animal house facility in a temperature (22 ± 2 °C) and humidity (30-70 %) controlled room (15 air changes/hour) with a 12:12 h light:dark cycles, had free access to rodent feed (Altromin Spezialfutter GmbH & Co. KG., Im Seelenkamp 20, D-32791, Lage, Germany) and water for one week before using for experimental purpose. Following 4 h fast (during the fasting period animals had free access to water) animals were divided into two groups (n=12/group). Group I animals (26-30 g) received ivosidenib orally at 5.0 mg/kg (suspension formulation comprising 0.5 % Tween-80 and methyl cellulose (0.5 % in water); strength: 0.5 mg/mL; dose volume: 10 mL/kg), whereas Group II animals (27-31 g) received ivosidenib intravenously [5 % DMSO, 5 % Solutol:absolute alcohol (1:1, v/v) and 90 % of normal saline; strength: 0.2 mg/mL; dose volume: 10 mL/kg] at 2.0 mg/kg dose. Post-dosing serial blood samples (40 μL, sparse sampling was done and at each time point three mice were used for blood sampling) were collected using Micropipettes (Microcaps®; catalogue number: 1-000-0500) through tail vein into polypropylene tubes containing K_2_EDTA solution as an anti-coagulant at 0.25, 0.5, 1, 2, 4, 8, 10, 12, 24, 30, 36 and 48 h (for oral study) and 0.12, 0.25, 0.5, 1, 2, 4, 8, 10, 12, 24 and 36 h (for intravenous study). Plasma was harvested by centrifuging the blood using Biofuge (Hereaus, Germany) at 1760 g for 5 min and stored frozen at -80 ± 10 °C until analysis. Animals were allowed to access feed 2 h post-dosing.

## Results and Discussion

### Liquid chromatography and MS/MS conditions

To avoid the potentially co-eluting peaks from blank plasma, which will influence the ionization efficiency of ivosidenib and the I.S, the mobile phase was optimized along with chromatographic conditions. In order to get chromatograms with good separation, peak shape and resolution for ivosidenib and the I.S, feasibility of various mixture(s) of solvents such as acetonitrile and methanol using different buffers such as ammonium acetate, ammonium formate and formic acid along with altered flow-rates (in the range of 0.8-1.2 mL/min) were tested. The resolution of ivosidenib and the I.S. was achieved with 0.2 % formic acid:acetonitrile (25:75, v/v) at a flow rate of 1.0 mL/min. Atlantis dC_18_ column (100 0 4.6 mm, 3 μm) was best suited to provide the symmetric peaks and baseline separation of ivosidenib and the I.S. with the retention time of 0.96 and 1.02 min, respectively. The total chromatographic run time was 2.0 min.

To obtain optimum ionization and sensitivity 100 ng/mL ivosidenib solution was directly injected into mass spectrometer and electrospray ionization (ESI) full scans were carried out both in positive and negative ion detection modes. Positive ESI mode was chosen as it provided good intensity signals and formed protonated [M+H]^+^ at *m/z* 583.1. This precursor ion (Q1) was selected for fragmentation in MS/MS mode to obtain most intense and consistent product ion (Q3: *m/z* 186.1) by optimizing the declustering potential, collision energy and collision cell exit potential. Subsequently, various gases like nebulizer gas (GS1), auxiliary gas (GS2), collision gas and source temperature were optimized to obtain adequate and reproducible response. MRM mode was used to obtain better selectivity, with dwell time of 100 ms for each transition. Fragment ions at *m/z* 476, 214, 186 and 158 were produced as prominent product ions for ivosidenib due to sequential loss of *m/z* 107, 262, 28 and 28 from ivosidenib. The postulated fragmentation pattern of ivosidenib is shown in Figure 1. Due to non-availability of deuterated ivosidenib to use it as an I.S, we have used warfarin as an I.S. and found to be the best for present purpose based on the chromatographic elution, ionization and reproducible and good extraction efficiency. For quantitation purpose *m/z* 583.1 precursor ion to the *m/z* 186.1 and *m/z* 309.2 precursor ion to the *m/z* 251.3 was used for ivosidenib and the I.S, respectively.

### Validation results

#### Selectivity

**Figure 2.**
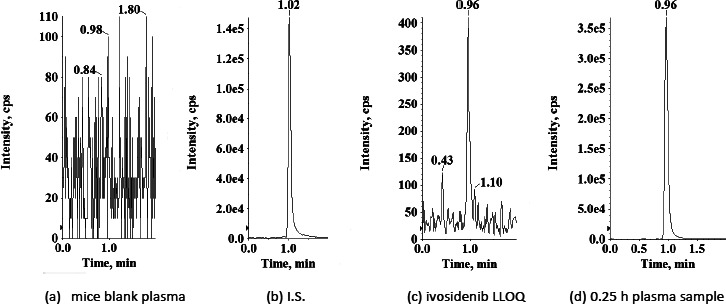
Typical MRM chromatograms of (a) mice blank sample (b) mice blank plasma spiked with I.S. (c) mice blank plasma spiked with ivosidenib at LLOQ (1.10 ng/mL) and (d) 0.25 h plasma sample showing ivosidenib (1953 ng/mL) peak obtained following 5.0 mg/kg oral dose of ivosidenib to mice.

Among the tested drug-free mice blank plasma samples no sample shown any interference at the retention times of the ivosidenib and the I.S. indicating that the method is selective (Figure 2).

#### Sensitivity and carry over

The lowest limit of reliable quantification for the analyte was set at the concentration of the LLOQ. The precision and accuracy at LLOQ concentration were found to be 6.12 and 91.0 % for ivosidenib. There was no carry-over produced by the highest calibration sample on the following injected blank plasma extract sample.

#### Recovery

The mean ± S.D recovery at LQC and HQC was found to be 66.5 ± 8.97 and 76.1 ± 1.43 %, respectively. The recovery of the I.S. was 100 ± 8.99 %.

#### Matrix effect

Six different lots of plasma samples, spiked with analyte concentration levels at LQC and HQC levels were analyzed. Matrix effect for ivosidenib at LQC and HQC was 0.99 ± 6.82 and 1.04 ± 1.70 %, respectively. The matrix effect for the I.S. was 1.09 ± 4.13%. The results have shown that the precision and accuracy for analyzed samples were within acceptance range. Overall it was found that there is no impact on the ionization of analyte and the I.S.

#### Calibration curve

The plasma calibration curve was constructed using eight calibration standards (viz., 1.10, 2.20, 21.9, 109, 548, 1097, 2264 and 3293 ng/mL). Under the analytical conditions used in the present study, the calibration curves of ivosidenib were found to be linear. Calibration curve was prepared by determining the best fit of peak-area ratios (peak area analyte/peak area IS) versus concentration, and fitted to the y = mx + c using weighing factor (1/X^2^). The average slope and intercept values were found to be 0.001173 and 0.00014, respectively. The average regression (n=4) was found to be ≥0.995. The lowest concentration with the RSD < 20 % was taken as LLOQ and was found to be 1.10 ng/mL. The % accuracy observed for the mean of back-calculated concentrations for four calibration curves for ivosidenib was within 90.8-110; while the precision (%RSD) values ranged from 2.08-9.42.

#### Accuracy and precision

A summary of the accuracy and precision data for intra- and inter-day is listed in Table 1. The assay values on both the occasions (intra- and inter-day) were found to be within the accepted variable limits.

#### Stability

The predicted concentrations for ivosidenib at 3.29 ng/mL (LQC) and 2607 ng/mL (HQC) samples in a battery of stability tests namely in-injector (24 h), bench-top (6 h), repeated three freeze/thaw cycles and freezer stability at -80 8 10 °C for 30 days are shown in Table 2. The results were found to be within the assay variability limits during the entire process.

#### Dilution effect

The accuracy and precision of the nominal concentration of the diluted plasma samples were within 0.79 % and 3.34 %, which show the ability to dilute samples up to a dilution factor of 10-fold in a linear fashion.

### Pharmacokinetic study

**Figure 3.**
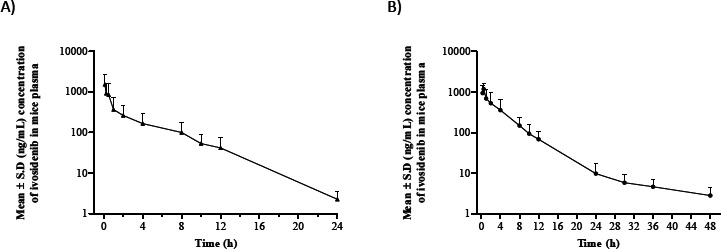
Mean plasma concentration-time profile of ivosidenib following (A) intravenous and (B) oral dosing to mice

The sensitivity and specificity of the validated assay was found to be sufficient for accurately characterizing the pharmacokinetics of ivosidenib in mice plasma following intravenous and oral administration. The estimates of the pharmacokinetic parameters are summarized in Table 3. The mean plasma concentration versus time profiles for ivosidenib following single oral and intravenous route is depicted in Figures 3A and 3B, respectively. Following intravenous administration, the plasma concentrations decreased in a mono-exponential manner. Ivosidenib was quantifiable up to 24 h post-dosing by intravenous route. The AUC_0-∞_ (area under curve from time zero to infinity) was found to be 4967 ng n h/mL. The in vivo clearance (CL) and volume of distribution (*V*_d_) was found to be 6.85 mL/min/kg and 1.70 L/kg, indicating that ivosidenib has low clearance and moderate volume of distribution. After oral administration of ivosidenib to mice, maximum concentration in plasma (*C*_max_: 1668 ng/mL) attained at 0.50 h (*T*_max_) indicating rapid absorption from gastrointestinal tract. Ivosidenib was quantifiable up to 36 h post oral dosing. The AUC_(0-∞)_ was found to be 7462 ng > h/mL. The terminal half-life was 2.87 and 4.06 h, by intravenous and oral routes, respectively. The absolute oral bioavailability was 61 %.

## Conclusions

A rapid, simple, sensitive and selective LC-ESI-MS/MS method was developed and validated for the determination of ivosidenib using 10 μL mice plasma. This method demonstrates good accuracy and precision and meets US FDA validation criteria. To our knowledge, this is the first report of an LC-MS/MS method for quantification of ivosidenib in plasma and successfully applied for a pharmacokinetic study in mice.

## Figures and Tables

**Table 1.** Precision, accuracy, recovery and matrix effect for determination of ivosidenib quality controls in mice plasma

	LLOQ QC (1.10 ng/mL)	LQC (3.29 ng/mL)	MQC (1715 ng/mL)	HQC (2607 ng/mL)
*Intra-day (n=6)*				
Mean ± S.D	1.09 ± 0.11	3.20 ± 0.28	1631 ± 96.2	2582 ± 188
Precision (%RSD)	9.91	8.86	5.72	7.31
Accuracy (%RE)	0.99	0.97	0.98	0.99
*Inter-day (n=24)*				
Mean ± S.D	1.10 ± 0.12	3.19 ± 0.27	1634 ± 96.4	2588 ± 188
Precision (%RSD)	10.7	8.48	5.90	7.25
Accuracy (%RE)	1.00	0.97	0.95	0.99

%RE: relative error (measured value/actual value ×100)

RSD: relative standard deviation (SD × 100/Mean)

SD: standard deviation

**Table 2.** Stability data of ivosidenib quality controls in mice plasma

Nominal concentration (ng/mL)	Stability	Mean ± SD^a^ n = 6 (ng/mL)	Accuracy (%)^b^	Precision (% RSD)
3.29	0 h 6 h (bench-top) 24 h (in-injector) Freeze-thaw 30 day at -80°C	3.21 ± 0.39 3.13 ± 0.40 2.98 ± 0.20 3.39 ± 0.50 3.32 ± 0.33	NA 97.6 92.9 106 103	12.1 12.6 6.87 14.8 10.1
2607	0 h 6 h (bench-top) 24 h (in-injector) Freeze-thaw 30 day at -80°C	2687 ±156 2709 ±76.4 2357 ±108 2632 ± 110 2609 ± 170	NA 101 87.7 98.0 97.1	4.78 2.82 4.57 4.18 6.50

^a^ Back-calculated plasma concentrations

^b^ (Mean assayed concentration / mean assayed concentration at 0 h) × 100

%RE: relative error (measured value/actual value × 100)

RSD: relative standard deviation (SD × 100/Mean)

**Table 3.** Pharmacokinetic parameters of ivosidenib in mice

Parameter	Intravenous	Oral
Dose (mg/kg)	2.0	5.0
AUC_0-∞_ (ng × h/mL)	4967	7462
*C*_0_/*C*_max_ (ng/mL)	6085	1668
*T*_max_ (h)	---	0.50
*t*_1/2_ (h)	2.87	4.06
*C*_L_ (mL/min/kg)	6.85	---
*V*_d_ (L)	1.70	---
